# Supplementary material for: The sizes of life
Source: PLoS One. 2023 Mar 29;18(3):e0283020. doi: 10.1371/journal.pone.0283020 (PMC10057745; doi:10.1371/journal.pone.0283020)
Supplement: S1 Fig — Grey dotted curves are 95% confidence bounds from 200 resamples from within-group uncertainties. See Fig 1 for color reference and default assumptions. A. Same data as main text, except with truncations at 1 log g on either side of reported minimum and maximum sizes. B. Same data as main text, except with truncations at reported minimum and maximum sizes. C. Sizes are defined for ramets or clones instead of genets, with truncation at -2 log g below the reported minimum size. D. Mass with low metabolism is omitted from body size and biomass estimates (plant woody material, hard coral skeleton, and subterranean microbes), with truncation at -2 log g below the reported minimum size. (PDF) [file pone.0283020.s001.pdf]

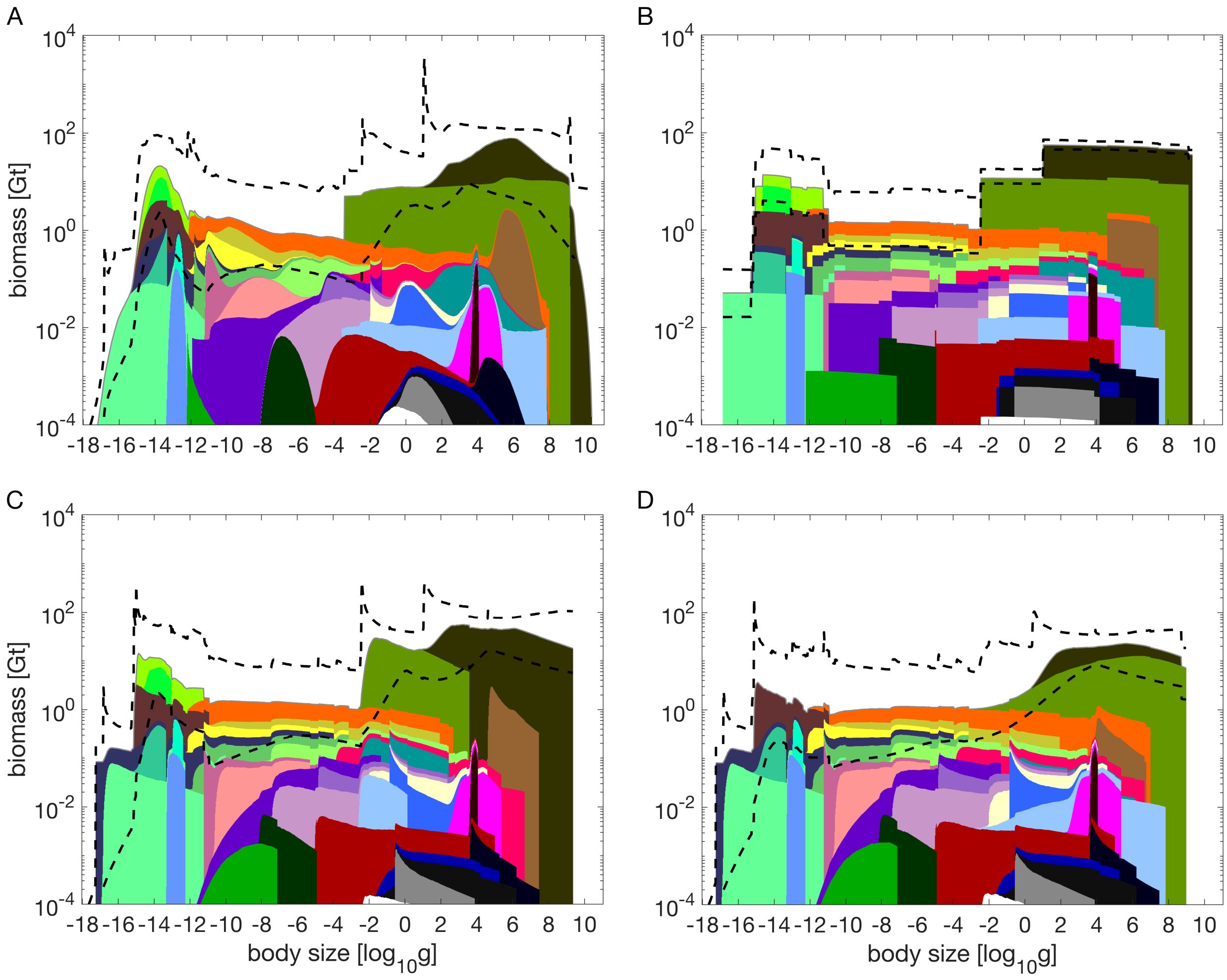

S1 Fig. Sensitivity of the global body size biomass spectrum to different assumptions. Grey dotted curves are 95% confidence bounds from 200 resamples from within-group uncertainties. See Fig. 1 for color reference and default assumptions. **A.** Same data as main text, except with truncations at 1 log g on either side of reported minimum and maximum sizes. **B.** Same data as main text, except with truncations at reported minimum and maximum sizes. **C.** Sizes are defined for ramets or clones instead of genets, with truncation at -2 log g below the reported minimum size. **D.** Mass with low metabolism is omitted from body size and biomass estimates (plant woody material, hard coral skeleton, and subterranean microbes), with truncation at -2 log g below the reported minimum size.
