# Supplementary material for: The sizes of life
Source: PLoS One. 2023 Mar 29;18(3):e0283020. doi: 10.1371/journal.pone.0283020 (PMC10057745; doi:10.1371/journal.pone.0283020)
Supplement: S2 Fig — Data is the same as in main text, except biomass is replaced by abundance or normalized biomass (biomass divided by size class width). Rows represent habitat realms (A: All realms, B: Terrestrial, C: Marine). Grey curves represent 95% confidence intervals of the data, and blue curves represent 95% confidence intervals of the model from 1000 bootstraps. α is the mean power exponent, and ± indicate standard deviations across bootstraps. Regression results are identical whether it is performed on log abundance or log normalized biomass as the dependent variable, because the latter is only offset from the former by a constant (-0.454). (PDF) [file pone.0283020.s002.pdf]

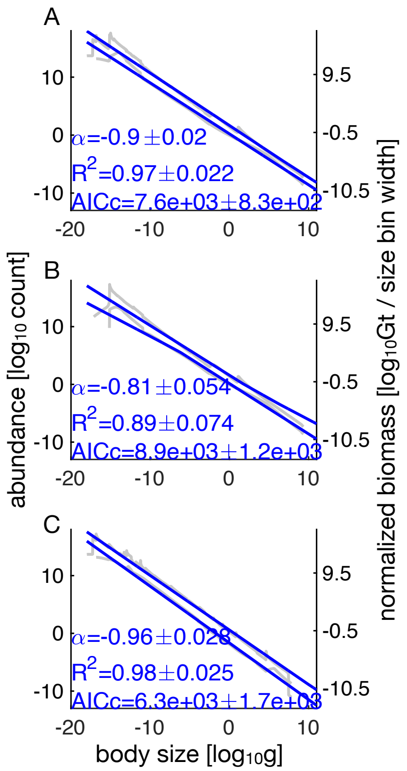

S2 Fig. Regression analyses on abundance. Data is the same as in main text, except biomass is replaced by abundance or normalized biomass (biomass divided by size class width). Rows represent habitat realms (**A**: all realms, **B**: terrestrial, **C**: marine). Grey curves represent 95% confidence intervals of the data, and blue curves represent 95% confidence intervals of the model from 1000 bootstraps. α is the mean power exponent, and $\pm$ indicate standard deviations across bootstraps. Regression results are identical whether it is performed on log abundance or log normalized biomass as the dependent variable, because the latter is only offset from the former by a constant (-0.454).
