# Supplementary material for: The sizes of life
Source: PLoS One. 2023 Mar 29;18(3):e0283020. doi: 10.1371/journal.pone.0283020 (PMC10057745; doi:10.1371/journal.pone.0283020)
Supplement: S3 Fig — Size ranges of 36 groups are quantified as the log max:min size ratio, corresponding to the number of log10 units that each group spans in size (g). This quantity shows no relationship with median body size (on log-log scale), with a power exponent of 0.0±0.10 (S.D.) and a p-value of 0.99. The size ratio has a mean of 7.0±4.2. (PDF) [file pone.0283020.s003.pdf]

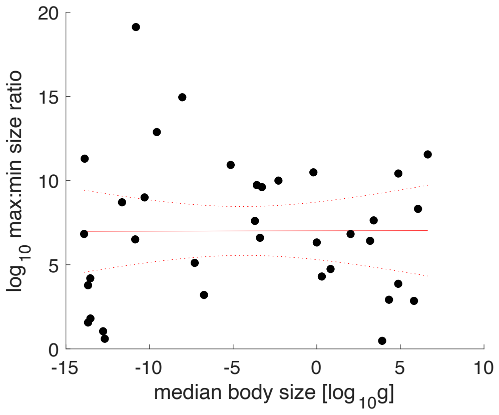

S3 Fig. Group size range. Size ranges of 36 groups are quantified as the log max:min size ratio, corresponding to the number of $\log_{10}$ units that each group spans in size (g). This quantity shows no relationship with median body size (on log-log scale), with a power exponent of 0.0±0.10 (S.D.) and a p-value of 0.99. The size ratio has a mean of 7.0±4.2.
