# Supplementary material for: The sizes of life
Source: PLoS One. 2023 Mar 29;18(3):e0283020. doi: 10.1371/journal.pone.0283020 (PMC10057745; doi:10.1371/journal.pone.0283020)
Supplement: S4 Fig — The size-biomass relationship for each group is composed of biomass and size estimates. Biomass estimates and uncertainties were mostly based on published syntheses that incorporate multiple independent sets of sampled biomass (black dots on maps) that are projected over habitat ranges (akin to species distribution models). Body size distribution and uncertainty were based on literature search for minimum, median, and maximum sizes within groups (green dots). A truncated generalized extreme value distribution was first fitted to the three points that result in an uncertainty estimate for median size. 1000 pairs of resampled total biomass and median size were then used to refit a truncated generalized extreme value distribution, resulting in a set of bootstrap samples that create the final median estimate and 95% confidence intervals for the size-biomass spectrum. (PDF) [file pone.0283020.s004.pdf]

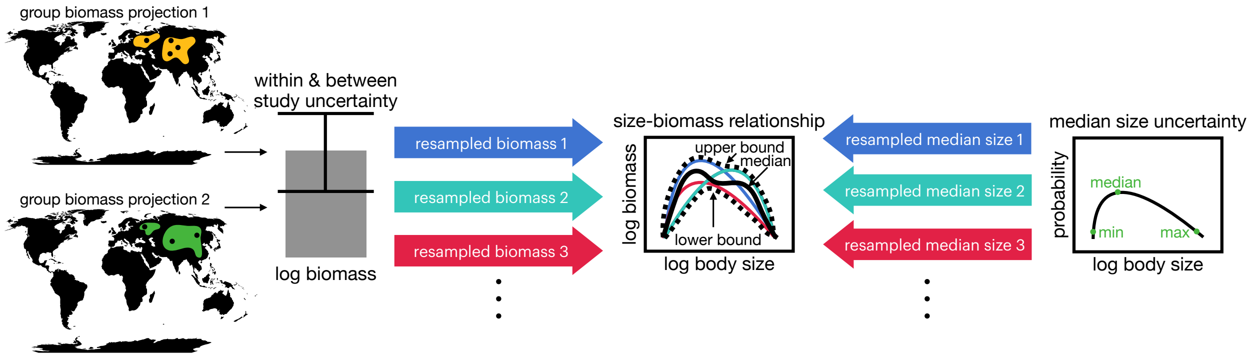

S4 Fig. Estimating within-group size-biomass spectrum. The size-biomass relationship for each group is composed of biomass and size estimates. Biomass estimates and uncertainties were mostly based on published syntheses that incorporate multiple independent sets of sampled biomass (black dots on maps) that are projected over habitat ranges (akin to species distribution models). Body size distribution and uncertainty were based on literature search for minimum, median, and maximum sizes within groups (green dots). A truncated generalized extreme value distribution was first fitted to the three points that result in an uncertainty estimate for median size. 1000 pairs of resampled total biomass and median size were then used to refit a truncated generalized extreme value distribution, resulting in a set of bootstrap samples that create the final median estimate and 95% confidence intervals for the size-biomass spectrum.
