# Supplementary material for: The sizes of life
Source: PLoS One. 2023 Mar 29;18(3):e0283020. doi: 10.1371/journal.pone.0283020 (PMC10057745; doi:10.1371/journal.pone.0283020)
Supplement: S1 Table — (PDF) [file pone.0283020.s005.pdf]

S1 Table. Body sizes measured for ramets instead of genets.

Group	Smallest body size	Largest body size	Min. body size (g C)	Median body size (g C)[a]	Max. body size (g C)	Biomass (Gt C)	Uncertainty (fold)
Grassland plants	no change	*Phyllostachys pubesces*	no change	3.76	3.78×10^{3} [1]	no change	no change
Seagrass	no change	*Posidonia oceanica*	no change	5.95×10^{-2}	1.35 [2]	no change	no change
Soil fungi	no change	*Phlebopus marginatus*	no change	1.88×10^{-5}	4.80×10^{2} [3]	no change	no change
Hard corals	*Madracis mirabilis*	*Mussa angulosa*	3.24×10^{-3} [4][b]	1.03	2.77×10^{2} [4][c]	no change	no change

[a] The median sizes for these affected groups were estimated as the geometric means of the new minimum and maximum sizes (resulting in a normal distribution on log size scale).

[b] Estimated as the skeleton density of weedy corals [5], multiplied by 0.653/0.603 (ratio of total coral biomass versus skeleton biomass) for size including tissue.

[c] Estimated as the skeleton density of stress-tolerant corals [5], multiplied by 0.653/0.603 (ratio of total coral biomass versus skeleton biomass) for size including tissue.
