# Supplementary material for: The sizes of life
Source: PLoS One. 2023 Mar 29;18(3):e0283020. doi: 10.1371/journal.pone.0283020 (PMC10057745; doi:10.1371/journal.pone.0283020)
Supplement: S2 Table — (PDF) [file pone.0283020.s006.pdf]

S2 Table. Body sizes excluding sizes and biomass with low metabolism.

Group	Smallest body size	Largest body size	Min. body size (g C)	Median body size (g C)	Max. body size (g C)	Biomass (Gt C)	Uncertainty (fold)
Forest plants	no change	no change	2.56	2.67×10^{5}	5.30×10^{8}	80[a]	no change
Grassland plants	no change	no change	2.67×10^{-3}	3.07×10^{6}	9.52×10^{8}	80[b]	no change
Mangroves	no change	no change	8.21×10^{3}	1.31×10^{5}	5.84×10^{6}	0.8[c]	no change
Hard corals	no change	no change	0.491	1.18×10^{2}	1.28×10^{6}	0.05[d]	no change
Subterranean bacteria	no change	no change	no change	no change	no change	0	no change
Subterranean archaea	no change	no change	no change	no change	no change	0	no change

[a] Mean allocation of plant materials to leaves and roots [6]. All sizes were scaled down by 80/337.5 (ratio of non-woody biomass versus total biomass).

[b] Mean allocation of plant materials to leaves and roots [6]. All sizes were scaled down by 80/112.5 (ratio of non-woody biomass versus total biomass).

[c] Estimated based on mangroves allocating a similar amount of material to leaves and roots as tropical plants [6]. All sizes were scaled down by 0.8/3.95 (ratio of non-woody biomass versus total biomass).

[d] Coral tissue biomass [7]. All sizes were scaled down by 0.05/0.653 (ratio of tissue biomass versus total biomass).
