# Supplementary material for: The sizes of life
Source: PLoS One. 2023 Mar 29;18(3):e0283020. doi: 10.1371/journal.pone.0283020 (PMC10057745; doi:10.1371/journal.pone.0283020)
Supplement: S3 Table — All icons belong to the public domain. (PDF) [file pone.0283020.s007.pdf]

S3 Table. Icon sources. All icons belong to the public domain.

Group	Icon source
Amphibians	Keynote
Wild birds	Keynote
Wild land mammals	Keynote
Reptiles	Keynote
Wild marine mammals	Keynote
Terrestrial nematodes	Custom
Marine nematodes	Custom
Cnidaria	Integration and Application Network, University of Maryland
Humans	Keynote
Livestock	Keynote
Seagrass	Integration and Application Network, University of Maryland
Bacterial picophytoplankton	Custom
Macroalgae	Integration and Application Network, University of Maryland
Molluscs	Keynote
Annelids	Pixabay.com
Terrestrial arthropods	Keynote
Phaeocystis	Custom
Green algae/protist picophytoplankton	Sally Bensusen, NASA EOS Project Science Office
Diatoms	Sally Bensusen, NASA EOS Project Science Office
Marine fungi	Custom
Marine archaea	Custom
Soil archaea	Custom
Hard coral	Custom
Fish	Keynote
Marine arthropods	Imgbin.com
Marine protists	Sally Bensusen, NASA EOS Project Science Office
Marine bacteria	Custom
Soil protists	Custom
Cryptogamic phototrophs	Kisscc0.com
Mangroves	Integration and Application Network, University of Maryland
Subterranean archaea	Custom
Soil bacteria	Custom
Soil fungi	Pixabay.com
Subterranean bacteria	Custom
Grassland plants	Keynote
Forest plants	Keynote
