# Supplementary material for: The sizes of life
Source: PLoS One. 2023 Mar 29;18(3):e0283020. doi: 10.1371/journal.pone.0283020 (PMC10057745; doi:10.1371/journal.pone.0283020)
Supplement: S1 File — (PDF) [file pone.0283020.s008.pdf]

S1 File. Supporting Information References.

1. Zhang H, Zhuang S, Sun B, Ji H, Li C, Zhou S. Estimation of biomass and carbon storage of moso bamboo (Phyllostachys pubescens Mazel ex Houz.) in southern China using a diameter–age bivariate distribution model. Forestry: An International Journal of Forest Research. 2014;87: 674–682. doi:10.1093/forestry/cpu028

2. Arnaud-Haond S, Duarte CM, Diaz-Almela E, Marbà N, Sintes T, Serrão EA. Implications of extreme life span in clonal organisms: Millenary clones in meadows of the threatened seagrass posidonia oceanica. PLoS ONE. 2012. doi:10.1371/journal.pone.0030454

3. Mcnabb RFR. The boletaceae of New Zealand. New Zealand Journal of Botany. 1968;6: 137–176. doi:10.1080/0028825X.1968.10429056

4. Precoda K, Hardt MJ, Baird AH, Madin JS. Tissue biomass trades off with growth but not reproduction in corals. Coral Reefs. 2020 [cited 25 May 2020]. doi:10.1007/s00338-020-01925-7

5. Darling ES, Alvarez-Filip L, Oliver TA, McClanahan TR, Côté IM. Evaluating life-history strategies of reef corals from species traits. Bellwood D, editor. Ecol Lett. 2012;15: 1378–1386. doi:10.1111/j.1461-0248.2012.01861.x

6. Poorter H, Niklas KJ, Reich PB, Oleksyn J, Poot P, Mommer L. Biomass allocation to leaves, stems and roots: meta-analyses of interspecific variation and environmental control: Tansley review. New Phytologist. 2012;193: 30–50. doi:10.1111/j.1469-8137.2011.03952.x

7. Bar-On YM, Phillips R, Milo R. The biomass distribution on Earth. Proceedings of the National Academy of Sciences. 2018; 201711842. doi:10.1073/pnas.1711842115
