# Supplementary material for: The sizes of life
Source: PLoS One. 2023 Mar 29;18(3):e0283020. doi: 10.1371/journal.pone.0283020 (PMC10057745; doi:10.1371/journal.pone.0283020)
Supplement: Attachment — Submitted filename: Reviewer 3 PONE ms 2022.docx [file pone.0283020.s009.docx]

Review of “The Sizes of Life”

E.W. Tekwa, Katrina A. Catalano, Anna L. Bazzicalupo, Mary I. O’Connor, Malin L. Pinsky

6 Jan 2023

Overall, I think the study should be worthy of publication and has the potential to be highly regarded and widely cited. Unfortunately, the way it is written does not endear the informed reader to the study. I think it oversells its novelty, fails to cite key prior work in some relevant places and omits discussion of its limitations and assumptions. I think these items are straightforward to address if the authors are willing and assuming these items can be fixed, I would recommend publication.

The paper claims that it remains unclear how biomass is distributed across body sizes (e.g. first sentence of abstract), and yet this has been extensively studied for over 50 years in aquatic systems and so is really only true in terrestrial systems. I think the paper should reserve its claims of novelty to the terrestrial realm and be more honest in its largely confirmatory status for aquatic systems. The paper needs to better delineate its actual contribution while acknowledging the extensive prior work in this field. Personally, I think the introductory remarks about energetics and size-density scaling at the species level could be replaced or expanded with a better treatment of the size spectrum in aquatic systems, concluding that very little has been done in terrestrial systems, and that this work fills that gap by confirming the aquatic results, and extending size distributions to terrestrial and truly global realms, albeit with extensive assumptions.

The paper has over 100 citations, but is light on highlighting prior work on the size spectrum. The paper also does not cite Bar-On et al. 2019 (its primary source of biomass data) or Hatton et al. 2021 (a prior global synthesis of marine systems) appropriately, IMO. Several sentence are also missing citations to prior work, despite explicit mention of such prior work (e.g. line 189, 227, 261, 278). Overall, the work would be better received by aquatic ecologists if the work can better integrate itself into the size spectrum literature.

Finally, although I was pleased with some of the supplementary analysis investigating the sensitivity of assumptions to results, it was difficult to evaluate in the context of how these assumptions might impact the overall findings. I think there should be a dedicated sub-section of the discussion that is more open about the limitations of the study. For example, how much do the results depend on the results of Bar-On et al., or the assumptions about within group size distributions?

I believe with a general toning down of the claims of novelty, a better treatment of prior work and a brief acknowledgements of the limitations, this paper would be acceptable and will be a valuable study. Below are listed a number of more specific suggestions that complement the three above-mentioned general areas for improvement.

Line 29: I think it is misleading to claim that the theories to explain Damuth’s law, or metabolic scaling have been “successful” – they continue to be debated and it seems safer to describe them as possible explanations.

Line 32: Size-density scaling of separate species with exponent ¾ is sometimes observed, and often not. If this is deemed relevant to the findings of this paper, it should be made clear that this is far from universal (and possibly due to underestimates on the slope due to OLS with ~3 orders of magnitude residual variation).

Line 35: I don’t believe it is well established that the size-spectrum slope of -1 is due to reduced energy transfer across trophic levels, as this sentence suggests. It may be reasonable, but not well established.

Line 46: The paper by Hatton et al. 2021 (Ref 21) is not properly cited. It is first cited in support of the dominance of medium sized plankton (which is incorrect), and later suggestive that they made misleading claims about the scope of their work. This study undertook analysis of the global marine size spectrum and should be cited appropriately, both acknowledging its precedence for the marine realm in the introduction and its results at line 117 and line 212.

Line 97: Nearly all biomass data are from Bar-On et al. 2019, and so this should be stated up-front in the introduction, since results should heavily depend on this single reference. It thus also does not appear to be cited appropriately until the Methods, which comes across as misleading. For example at line 72 (“The resulting catalogue of biomass data matched to body sizes stands as a record of present knowledge about life on Earth”), it seems to be misleading not to cite the source for the biomass data. It is also the primary source for one of the three major conclusions.

Line 190: The discussion lists three major biological findings, the first of which I did not understand (unless it is the trivial finding that different taxonomic groups can have similar body sizes), the second of which is confirmatory of prior work going back to Sheldon et al. 1972 (at least in aquatic systems), and the third of which is a restatement of prior work such as Bar-On et al. 2019. These and other references to prior work need to be explicitly acknowledged.

The paragraph starting at line 197 makes some poorly justified claims, in my opinion. On one hand, the range of 10^7 to 10^9 (two decades) is considered a narrow range, while on the other hand, further down the paragraph, the roughly one order of magnitude greater biomass at the extremes of the size spectrum is considered significant (despite the considerably greater uncertainty in these estimates). Both claims (coincidence in max body sizes and significant internal modes) appear quite weak in my opinion.

The paragraph starting at line 220 makes a cursory attempt to explain the differences between terrestrial and aquatic size spectra. Despite the introduction of the paper and this paragraph itself making mention of the energetic considerations that have dominated this literature, there is no mention of the presence of woody, non-metabolically active material. It is excellent that the authors undertook this analysis (Fig. S3 D) and I think it should be mentioned as a contributing factor to the hump, though possibly not sufficient to account for it. This gives more credence to a possible energetic basis for the origin of the size spectrum. At least I would expect the authors to make mention of woody material in this paragraph (in addition to line 255), and cite their SM analysis.

Methods

Perhaps I am not understanding the methods, but fitting three points (min, median and max) to a three parameter distribution does not inspire confidence, especially when these input data are so uncertain and each group somewhat arbitrary. I am thus not convinced that any hump in the distribution is significant, and so don’t see a strong justification that non-linear methods should be superior to a linear fit on log-axes (line 270).

Line 325: What is the reference for “assuming a log-normal size-biomass distribution”?

Line 330: Assuming sizes at maturity are representative of a group are particularly problematic for fish and trees, where “maturity” may be several orders of magnitude larger than juveniles and could skew results noticeably (even on log scales).

Line 365: I did not understand the sentence beginning at this line.

Line 457: I did not understand this paragraph.

Table S2: I did not understand this table. I think this is a valuable analysis and so some further description is warranted.

I wish the authors the best of luck in addressing my concerns.
